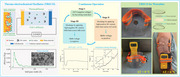# Self‐powered wearable medical devices for cognitively impaired older adults

**DOI:** 10.1002/alz70858_103821

**Published:** 2025-12-26

**Authors:** Morteza Sabet, Basanta Ghimire, Mihir Parekh, Herbert Behlow, Sriparna Bhattacharya, Zahra Rahemi, Apparao M Rao

**Affiliations:** ^1^ Clemson University, Greenville, SC, USA; ^2^ Clemson University, Clemson, SC, USA

## Abstract

**Background:**

The demand for wearable medical devices among older adults, particularly those with cognitive decline, is growing. Developing self‐powered wearables that eliminate the need for battery recharging or replacement will significantly enhance user‐friendliness for individuals with cognitive decline, who may forget to maintain their devices. Constant, passive power from natural sources may be key to unlocking wearables’ potential. The heat produced by human bodies, as a natural and sustainable energy source, is a prime candidate for this task. The use of thermoelectric generators (TEGs) for harvesting high‐grade heat has been extensively studied over time. However, capturing ubiquitous low‐grade heat near room temperature (e.g., body heat) remains a significant challenge.

**Method:**

We developed a thermally rechargeable electrochemical oscillator (TRECO) that harvests ultralow‐grade heat for powering small devices. Specifically, an ionic thermoelectric cell composed of two porous electrodes and a cellulosic separator was assembled and evaluated. The voltage generation near room temperature based on the Soret effect was studied for several porous electrodes.

**Result:**

The armband with a single TRECO cell generated approximately 825 mV from a small temperature difference of 6 K, with human skin as the hot end and the surrounding environment as the cold end. This results in a more than 130 mV K^‐1^ thermopower, almost four to five times the record to date. It was found that the electrolyte composition and the electrode porosity and microstructure can influence the device's thermopower. The developed device can be charged by maintaining a low‐temperature difference across its two electrodes.

**Conclusion:**

A novel ionic thermoelectric device was developed to harvest ultralow‐grade heat from the body for power generation effectively. This technology holds great potential for powering wearable health monitoring devices for older adults with cognitive decline, as it can overcome challenges associated with their cognitive impairment, which may hinder their ability to recharge their devices.